# *Msh2* deficiency leads to dysmyelination of the corpus callosum, impaired locomotion, and altered sensory function in mice

**DOI:** 10.1038/srep30757

**Published:** 2016-08-01

**Authors:** Barthelemy Diouf, Prakash Devaraju, Laura J. Janke, Yiping Fan, Sharon Frase, Donnie Eddins, Jennifer L. Peters, Jieun Kim, Deqing Pei, Cheng Cheng, Stanislav S. Zakharenko, William E. Evans

**Affiliations:** 1 Hematological Malignancies Program and Department of Pharmaceutical Sciences, St. Jude Children’s Research Hospital, Memphis, 38105, USA; 2 Department of Developmental Neurobiology, St. Jude Children’s Research Hospital, Memphis, 38105, USA; 3 Department of Pathology, St. Jude Children’s Research Hospital, Memphis, 38105, USA; 4 Department of Computational Biology, St. Jude Children’s Research Hospital, Memphis, 38105, USA; 5 Cell and Tissue Imaging Resource, St. Jude Children’s Research Hospital, Memphis, 38105, USA; 6 Small Animal Imaging Center, St. Jude Children’s Research Hospital, Memphis 38105, USA; 7 Department of Biostatistics, St. Jude Children’s Research Hospital, Memphis 38105, USA

## Abstract

A feature in patients with constitutional DNA-mismatch repair deficiency is agenesis of the corpus callosum, the cause of which has not been established. Here we report a previously unrecognized consequence of deficiency in MSH2, a protein known primarily for its function in correcting nucleotide mismatches or insertions and deletions in duplex DNA caused by errors in DNA replication or recombination. We documented that *Msh2* deficiency causes dysmyelination of the axonal projections in the corpus callosum. Evoked action potentials in the myelinated corpus callosum projections of *Msh2-*null mice were smaller than wild-type mice, whereas unmyelinated axons showed no difference. *Msh2-*null mice were also impaired in locomotive activity and had an abnormal response to heat. These findings reveal a novel pathogenic consequence of MSH2 deficiency, providing a new mechanistic hint to previously recognized neurological disorders in patients with inherited DNA-mismatch repair deficiency.

The integrity of cellular DNA is continually challenged by replication errors, thereby potentially altering the sequence or chemical composition of DNA. Once detected, these lesions can be repaired by one of several protein complexes responsible for repairing DNA-base mismatches, insertions/deletion, or errors that occur during DNA replication^1^.

Defects in cellular DNA-repair processes have been linked to genome instability and several human cancers^2^,^3^. More recently, deficiency in DNA repair has been linked to several neurodegenerative diseases, such as ataxia telangiectasia, spinocerebellar ataxia with axonal neuropathy, and ataxia with oculomotor apraxia^4^. However, the mechanisms by which deficiency in DNA repair genes cause neurodegenerative diseases have remained largely unknown^5^.

Oligodendrocytes are a class of glial cells that wrap around neuronal axons to form myelin sheaths that facilitate the rapid propagation of action potentials^6^,^7^. The integrity of myelin is essential for normal functioning of the central nervous system (CNS); therefore, illnesses that involve myelin damage can have devastating consequences^8^. Defective myelination can be caused by demyelination, which is characterized by reduced levels of myelin following neurodegeneration and neuronal loss, or dysmyelination, which is characterized by a failure of oligodendrocyte differentiation and myelin synthesis^9^,^10^. The molecular bases of oligodendrocyte differentiation and CNS myelination are still incompletely understood. However, the identification of new transcriptional regulators of myelin gene expression and the discovery of signaling pathways that orchestrate the development of myelin have provided important new mechanistic insights^11^. It has also been shown that the RNA binding protein QKI (quaking protein) plays a crucial role in myelinogenesis by regulating myelin-specific genes^12^.

MSH2 is a component of the DNA-mismatch repair (MMR) system that plays an essential role in correcting replication errors resulting in incorporation of the wrong nucleotide or causing nucleotide deletions or insertions. The human gene encoding MSH2 exhibits genetic polymorphism, and inheritance of some variant forms of *MSH2* predisposes to the development of multiple types of cancers (mainly colon and endometrial cancers) and causes drug resistance in leukemia^13^,^14^,^15^,^16^. A recently discovered common feature among patients with constitutional mismatch repair deficiency (CMMR-D) is agenesis of the corpus callosum, but the underlying mechanism of this neurologic defect is unknown^17^. CMMR-D, a rare inherited syndrome that predisposes children to cancer, is caused by biallelic germline variants in one of four genes that control DNA MMR: *MSH2, MSH6, MLH1* and *PMS2*^17^.

Here we show that disruption of the MMR gene *Msh2* leads to low *Qki* expression and dysmyelination in the corpus callosum of mice. Furthermore, we show that the dysmyelinated axons in *Msh2*-deficient corpus callosum generate abnormal action potentials. The *Msh2*-deficient animals display abnormal locomotor activity and sensitivity to heat. These findings reveal a previously unrecognized consequence of MSH2 deficiency and provide mechanistic insight into the basis of corpus callosum agenesis in patients with constitutional mismatch repair deficiency.

## Results

### *QKI* expression after *MSH2* deficiency

**Figure 1:**
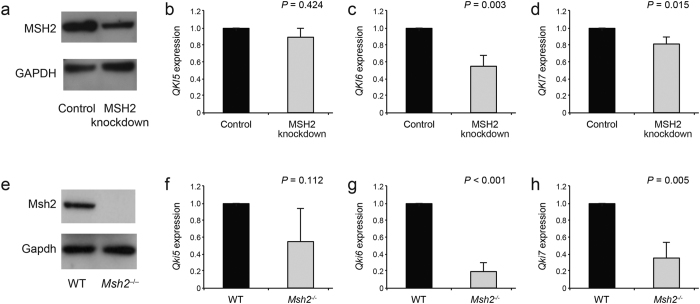
Expression of QKI is significantly lower in the absence of MSH2 expression. (**a**) *MSH2* was knocked down in human leukemia cells (CEM cell line), and the mRNA expression of *QKI* isoforms was quantified by Real-time PCR and normalized to the *GAPDH* expression (**b,c,d**). The mRNA expression of *Qki* isoforms was also quantified by Real-time PCR and normalized to *Gapdh* expression in the corpus callosum of *Msh2*^−/−^ mice (**e,f,g,h**). Panels (**a**,**e**) represent western blots. Error bars represent SD. Data represent triplicate experiments in the CEM cell line and n = 3 mice in each genotype. The blots have been run under the same experimental conditions.

We assessed by quantitative real-time PCR the differences in gene expression after knock down of *MSH2* in CEM human leukemia cells. This revealed that the level of expression of the *QKI* transcript (particularly *QKI6* and *QKI7*) was markedly lower after *MSH2* knock down (Fig. 1a–d) (p = 0.003, 95% confidence interval of difference in mean [−0.6 to −0.31] for *QKI6* and *p* = 0.015, 95% confidence interval of difference in mean [−0.28 to −0.1] for *QKI7*). Markedly lower expression of *Qki* was also documented in the corpus callosum of *Msh2*^−/−^ mice (Fig. 1e–h) (*p* < 0.001, 95% confidence interval of difference in mean [−0.91 to −0.7] for *Qki6* and *p* = 0.005, 95% confidence interval of difference in mean [−0.83 to −0.4] for *Qki7*). QKI is an RNA-binding protein that controls the translation of genes involved in oligodendrocyte differentiation^18^.

### Myelin defects in the corpus callosum of *Msh2* ^−/−^ mice

Because oligodendrocytes are the myelin-forming glial cells in the CNS^19^, we used electron microscopy to assess myelin sheath structure around axonal projections in the corpus callosum of *Msh2*^−/−^ mice. This fibrous brain structure is normally enriched with myelinated axons because it is the main point at which important signals are integrated and transferred between the two hemispheres of the brain^20^.

**Figure 2:**
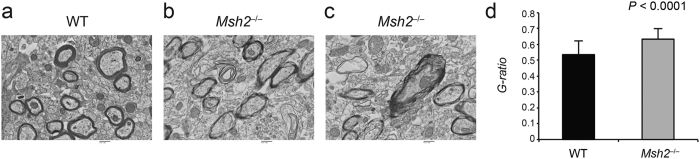
Myelin ultrastructure in the corpus callosum of the *Msh2*^−/−^mice. Transmission electron microscopic images of cross-sections at high magnification show disorganized myelin in the *Msh2*^−/−^ mice (**b,c**) compared to normal, compact myelin in the wild-type (WT) mice (**a**). Scale bars represent 500 nm. (**d**) Determination of myelin sheath thickness by G-ratio quantification from 50 axons in each mouse. Error bars represent SD. N = 3 mice for each genotype.

Electron microscopic analysis revealed loosely wrapped myelin surrounding the axons in the *Msh2*^−/−^ mice compared to the compact myelin sheaths observed in wild-type (WT) mice (Fig. 2a–c). The calculations of the G-ratios for the myelinated axons in the corpus callosum showed a significant difference between WT and *Msh2*^−/−^ mice (Fig. 2d) (p < 0.0001, 95% confidence interval of difference in mean [0.1 to 0.11]. Axon diameter was not significantly different between WT and *Msh2*^−/−^ mice (Supplementary Fig. S1). These results suggest that *Msh2* deficiency leads to dysmyelination due to impaired oligodendrocyte differentiation and myelin synthesis^9^.

### Dysmyelination not demyelination in *Msh2* ^−/−^ mice

Additional studies were performed to rule out other potential causes of defective myelination, including mechanisms of demyelination^10^. In the case of multiple sclerosis, demyelination is caused by an autoimmune attack on myelin and oligodendrocytes^21^ and occurs, in part, as a consequence of inflammatory cells infiltrating the CNS^22^. To rule out this having occurred in *Msh2*^−/−^ mice, we immunostained the corpus callosum with anti-CD3 antibody, which documented the absence of T cell infiltrates in this structure of the *Msh2*^−/−^ mice (Supplementary Fig. S2).

**Figure 3:**
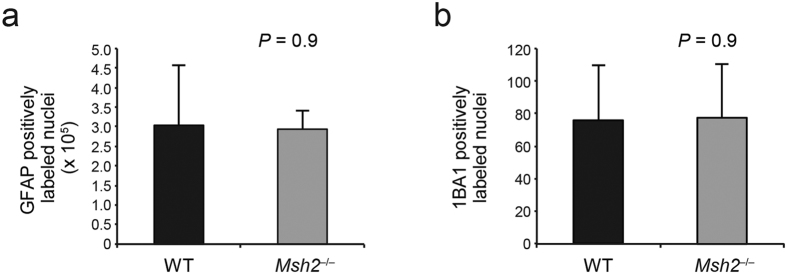
Immunohistochemical labeling. (**a**) Paraffin section were stained using an antibody anti GFAP. The quantification of the results showed no difference between WT and *Msh2*^−/−^ mice. Data are represented as means ± SD (n = 4 for each genotype). (**b**) Paraffin section were stained using an antibody anti IBA1. The quantification of the results showed no difference between WT and *Msh2*^−/−^ mice. Data are represented as means ± SD (n = 4 for each genotype).

Astrocytosis can also lead to myelin abnormalities^23^, but immunostaining for the glial fibrillary acidic protein (GFAP), an astrocyte marker, did not reveal any features associated with astrocytosis in the *Msh2*^−/−^ mice (Fig. 3a and Supplementary Fig. S3) (*p* = 0.9). Microglia can proliferate and acquire activated morphologies in conditions of neuronal and glial damage^24^. Thus, we tested the expression of Iba-1, a calcium-binding protein commonly used to assess the status of microglial activation^25^. Immunostaining for Iba-1 did not reveal activated microglia in the corpus callosum of the *Msh2*^−/−^ mice (Fig. 3b, Supplementary Fig. S4) (*p* = 0.9). Immunostaining for cleaved caspase 3 also showed no difference between the *Msh2*^−/−^ mice and WT littermates, indicating that myelin defects in the *Msh2*^−/−^ mice are not caused by increased caspase 3–mediated apoptosis of oligodendrocytes or other cells (Supplementary Fig. S5).

**Figure 4:**
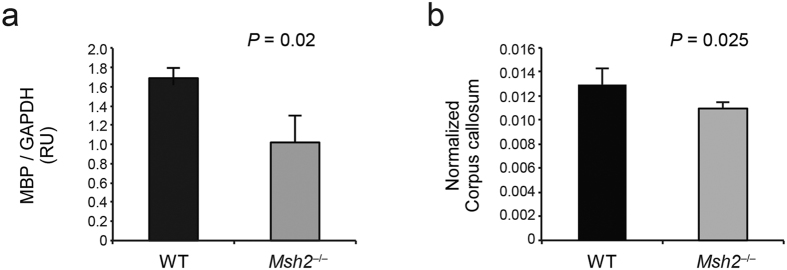
MBP expression and *in vivo* MRI. (**a**) Quantification by densitometry of all the isoforms of MBP protein levels in the corpus callosum normalized to GAPDH signal shows low expression of myelin basic protein (MBP) in the *Msh2*^−/−^ mice compared to that in WT mice. Error bars represent SD (n = 3 for each genotype). (**b**) Corpus callosum volumes determined by MRI and normalized to brain volume were significantly smaller in the *Msh2*^−/−^. Error bars represent SD (n = 4 for each genotype).

Myelination of neurons in the CNS depends on the correct execution of a genetic program of oligodendrocyte differentiation that culminates in the timely, coordinated expression of several myelin genes^26^. To assess deficits in oligodendrocyte differentiation and myelin formation, we compared the expression of myelin basic protein (MBP), a protein related to myelination, in the corpus callosum of WT mice and *Msh2*^−/−^ mice by western blot. MBP protein level normalized by GAPDH was significantly lower in the corpus callosum of the *Msh2*^−/−^ mice than in WT littermates (Fig. 4a and supplementary Fig. S6) (*p* = 0.02, 95% confidence interval of difference in mean [−0.78 to −0.44]), supporting dysmyelination rather than demyelination as a primary pathogenic consequence of MSH2 deficiency. These results are consistent with the altered expression of myelin associated glycoprotein (MAG), a target of QKI^27^ (Supplementary Fig. S7).

To determine if dysmyelination affects the volume of the corpus callosum we performed *in vivo* magnetic resonance imaging (MRI). This documented that *Msh2*^−/−^ mice have a significantly smaller corpus callosum compared to the WT mice, after normalizing corpus callosum volume to brain volume. (Fig. 4b, Supplementary Fig. S8) (*p* = 0.025).

### Evoked electrophysiological responses in the corpus callosum of *Msh2* ^−/−^ mice

**Figure 5:**
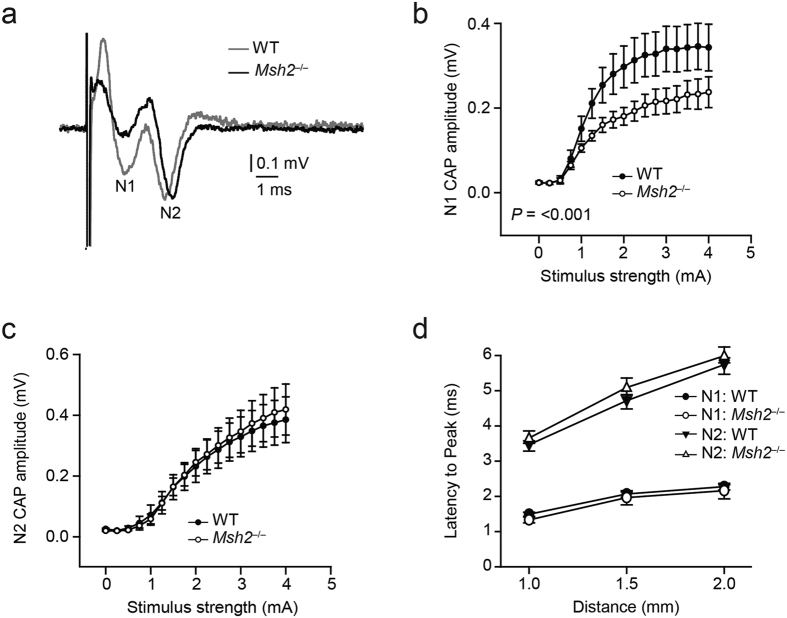
Electrophysiological deficits in *Msh2*^−/−^ mice. (**a**) Representative corpus callosal compound action potential (CAP) traces from a WT mouse showing typical N1 and N2 components (gray line) compared to a smaller amplitude N1 component in a *Msh2*^−/−^ mouse (black line). (**b**) Quantification of the average stimulus-response (WT, n = 12 slices, 4 mice; *Msh2*^−/−^, n = 13 slices, 4 mice) revealed a significant decrease in the amplitude of the N1 component (myelinated axons) in the *Msh2*^−/−^ mice (*P *< 0.001). (**c**) There was no difference in the amplitude of the N2 (unmyelinated axons) component. (**d**) N1 and N2 conduction velocities, which were represented by the respective latencies to peak at different distances (1–2 mm), revealed no difference between the WT and the *Msh2*^−/−^ mice. Electrophysiological data are represented as the mean ± SEM.

The myelin sheath, which is composed of multilamellar plasma membranes from oligodendrocytes, is crucial for neuronal function as it effectively propagates action potentials along axons^28^,^29^. To evaluate the functional consequences of Msh2 deficiency in mice, we performed electrophysiological analyses of compound action potentials (CAPs) evoked in the corpus callosum. CAPs have been previously characterized in the mouse corpus callosum as a biphasic waveform with an early component (1- to 2-ms latency) representing fast depolarization from mostly large myelinated axons (N1) and a later component (3- to 6-ms latency) representing slower depolarization from nonmyelinated axons (N2)^30^. The electrically evoked CAPs in the corpus callosum of WT mice and *Msh2*^−/−^ mice had typical N1 and N2 components that could be separated by their latencies (Fig. 5a). Quantification of the average stimulus-evoked response showed that the N1 amplitude was significantly smaller in the *Msh2*^−/−^ mice than in WT littermates (0.25 ± 0.03 mV vs. 0.34 ± 0.03 mV, *p* < 0.001) and it increased significantly slower in the *Msh2*^−/−^ mice at the stimulus strength 1mA, (95% confidence interval of difference in mean slope [−0.218 to −0.023]) (Fig. 5b). In contrast, the N2 amplitude showed no difference (0.39 ± 0.05 mV in WT vs. 0.42 ± 0.05 mV in *Msh2*^−/−^ *p* = 0.61) at 4 mA-stimulation (Fig. 5c). Thus, *Msh2* deficiency affects myelinated but not unmyelinated axons. Despite the reduced N1 amplitude, we did not observe any significant difference in the conduction velocities associated with either myelinated or unmyelinated axons, suggesting that the speed of propagation of action potentials in *Msh2*^−/−^ mice is unaffected (N1 latency at 1.5-mm distance: 2.06 ± 0.08 ms in WT vs. 1.97 ± 0.08 ms in *Msh2*^−/−^, *p* = 0.5; N2 latency at 1.5-mm distance: 4.71 ± 0.09 ms in WT vs. 5.09 ± 0.08 ms in *Msh2*^−/−^, *p* = 0.17) (Fig. 5d). Normal propagation velocities and a decrease in N1 amplitude are consistent with dysmyelination as the cause of deficient axonal excitability in *Msh2*^−/−^ mice.

### Behavioral alterations in the *Msh2* ^−/−^ mice

**Figure 6:**
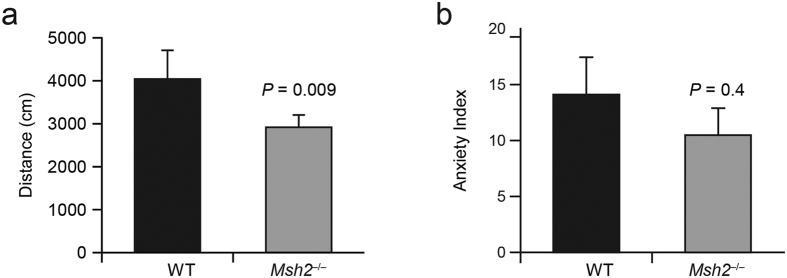
Impaired locomotion and sensation in *Msh2*^−/−^ mice. (**a**) Mice were placed in an open-field chamber equipped with infrared sensors. Total distance traveled (in centimeters) was measured for each group of five mice. *Msh2*^−/−^ mice walked a significantly shorter distance than did the WT mice. Error bars represent SD (n = 5 for each genotype). (**b**) Mice were placed in an open-field chamber equipped with infrared sensors. Anxiety index is the ratio between the time in the center and the time in the periphery (n = 5 for each genotype).

**Figure 7:**
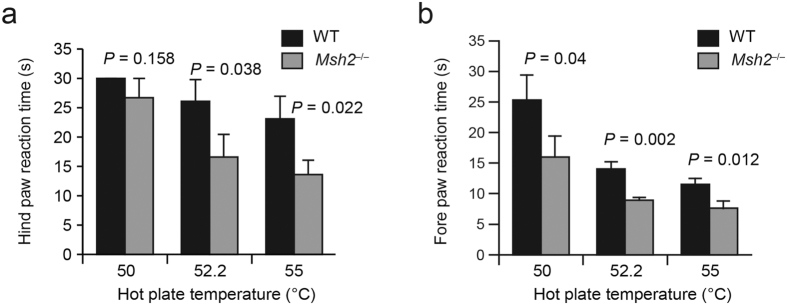
Altered sensation in *Msh2*^−/−^ mice. The hot-plate test was performed at 50 °C, 52.5 °C, and 55 °C. The time(s) elapsing to the first pain response (lifting or licking or jumping the hind paws (**a**) or the fore paws (**b**)) was scored for the WT (solid black) and the *Msh2*^−/−^ (grey) mice. 3 mice for each genotype were tested. Behavioral data are represented as means ± SD.

Mouse mutants with dysmyelination can exhibit impaired motor function^31^. Furthermore agenesis of the corpus callosum was associated to motor and sensory neuropathy in different studies^32^,^33^. To assess whether Msh2 deficiency has behavioral consequences, we used an open-field test to compare locomotor activity in *Msh2*^−/−^ mice and WT mice. The total distance traveled by *Msh2*^−/−^ mice was significantly less than that of WT littermates (n = 5 for each genotype; Fig. 6a) (p = 0.009, 95% confidence interval of difference in mean [−1873.0 to −375.0]), which was not due to a difference in anxiety (Fig. 6b). The hot plate test was used to determine whether *Msh2*^−/−^ mice also have altered sensory perception, revealing that the minimum time of response at different temperatures was shorter for *Msh2*^−/−^ mice compared to *Msh2* WT mice (Fig. 7a,b) (p = 0.038, 95% confidence interval of difference mean [−18.073 to −0.861] at 52.5 °C; *p *= 0.023, 95% confidence interval of difference in mean 16.869 to −2.197] at 55 °C).

## Discussion

By showing that *Msh2* deficiency is associated with myelin defects, we have revealed previously unrecognized consequences of MSH2 deficiency, a protein that has been extensively characterized for its role in DNA mismatch repair and the increased risk of several types of human cancers in patients who inherit or acquire loss of function variants in *MSH2*. Our findings provide mechanistic insights to the growing body of evidence linking DNA repair and neurodegeneration, with at least 16 hereditary DNA-repair diseases for which neurologic abnormalities represent a major^4^,^5^,^34^, and in some cases the only, clinical consequence of mutations in DNA-repair genes^10^.

The interaction of neurons and glial cells is a feature of virtually all nervous systems^35^. Although oligodendrocytes generate myelin, other glial cells, including astrocytes and microglia, can also affect myelin. The activation of microglia (microgliosis) is frequently accompanied by that of astrocytes (astrogliosis)^24^. Once activated, these glial cells can contribute to degenerative processes by releasing various neurotoxic cytokines^36^. In some myelin disorders such as Alexander disease and vanishing white matter disease, myelin loss may be caused by astroglial pathology^37^. Here we show that *Msh2*^−/−^ mice did not exhibit astrocytosis or microgliosis. Moreover, *Msh2* deficiency did not cause T-cell infiltration or caspase 3–mediated apoptosis, both of which can indirectly cause myelin abnormalities. However, *Msh2* deficiency was associated with low *Qki* expression, which is consistent with dysmyeliniation^38^. Electron microscopy of the corpus callosum confirmed dysmyelination in *Msh2*^−/−^ mice. Lower corpus callosum volume has been shown to be associated with the severity of disease characterized by dysmyelination^39^,^40^, which is in accordance with our findings. The preponderance of studies of the *MSH2* gene have focused on its role in DNA repair, with few mechanistic insights to explain the observed relation between DNA repair and neurodegeneration. Dysmyelination has been attributed to a deficiency in DNA repair and to defective gene transcriptional regulation^9^,^10^. The mechanism for low *Qki* expression that we observed in Msh2-deficient mice and in human cells in which MSH2 expression was knocked down is unclear, and merits further study based on the current finding of a relationship between MSH2-deficiency and dysmyelination.

The myelin sheath is required for fast propagation of action potentials along axons^31^, and damage of the myelin sheath is a well-known characteristic of many neurological disorders^41^. Here we assessed the generation and propagation of action potentials in the corpus callosum by using an electrophysiological method widely used to characterize white matter dysfunction following myelin defects^30^,^42^. This revealed a significant decrease of amplitude of action potentials evoked in myelinated (fast) but not in unmyelinated (slow) axons in *Msh2*^−/−^ mice, consistent with axonal integrity requiring an intact myelin sheath^28^. Some neurological diseases affect the functional integrity of axons ensheathed by defective myelin^35^, and some myelin disorders result in axonal degeneration. Although the absence of myelin does not always result in secondary axonal defects, the fact that action potential amplitudes were normal in the unmyelinated axons in *Msh2*^−/−^ mice speaks for dysmyelination occurring prior to axonal dysfunction. This conclusion is supported by previous work showing that dysmyelination can cause a deficiency in axonal function^25^.

The myelin sheath is required for proper functioning of most long-range axonal projections involved in motor or sensory functions of the brain. *Msh2*^−/−^ mice do not exhibit any obvious gross behavioral abnormalities; however, *Msh2*^−/−^ mice have significantly altered motor and sensory functions as documented by the open field and hot plate tests. This is consistent with the importance of proper myelination of axonal projections for normal sensation and motor functions^31^. This is also consistent with the association between neuropathy and absence or malformation of the corpus callosum^32^,^33^. Although the current work has documented that MSH2 deficiency leads to dysmyelination in the corpus callosum, it is plausible that MSH2 deficiency also affects myelin biogenesis in other tissues as well.

MSH2 belongs to the DNA MMR system. The initiation of the repair process depends on mismatch recognition by the heterodimeric protein complex MutSα (composed of MSH2 and MSH6) or MutSβ(composed of MSH2 and MSH3)^43^. It would be interesting to determine whether dysmyelination is specific to loss of MSH2 or is evident with the loss of other DNA mismatch repair proteins or whether concomitant loss of multiple MMR genes has an even greater effect on the corpus callosum.

In conclusion, we show that the corpus callosum of *Msh2*-deficient mice contains dysmyelinated axonal projections, and this defect is associated with abnormalities in action potentials, locomotion, and sensation. These findings establish a new link between a DNA-repair protein and neuronal and behavioral deficits, thereby providing new mechanistic insight to neurological defects associated with diseases such as constitutional mismatch repair deficiency.

## Methods

### Animals

All animal studies were approved by the St. Jude Animal Care and Use Committee. The methods were carried out in accordance with the approved guidelines. C57BL/6 males mice (aged 6–8 weeks) were used. *Msh2*^−/−^ mice and WT littermates were generously provided by Dr. Tak Mak (Amgen Institute, Toronto, ON, Canada)^15^. The mice were genotyped by a previously described polymerase chain reaction method^44^.

### Quantitative Real-Time PCR (qRT-PCR)

Total RNA from CEM cells and from mouse corpus callosum were isolated using TRIzol reagent according to the manufacturer’s instructions. RNA was treated with DNase I and was reverse transcribed using oligo-dT primer and Superscript III according to the protocol of the manufacturer. Real-time PCR was performed with the cDNAs using SyBR Green PCR Master Mix (according to the Applied Biosystems instructions) on a 7900HT Real-time PCR System (Applied Biosystems). The qRT-PCR conditions were as follow: 95 °C for 10 minutes followed by 40 cycles of 15 seconds at 95 °C and 1min at 60 °C. Gene expression was quantified using cycle threshold (Ct) values and normalized to GAPDH (ΔCT). The quantitative relative expression was determined using 2^−ΔΔCT^ with gene expression in *Msh2*^−/−^ mice expressed as relative to gene expression in WT mice and gene expression in *knockdown* CEM cells relative to gene expression in Control CEM cells. For the Real-time PCR, the following primers were used: Mouse and Human QKI5 forward primer 5-CTGTCATGCCAAACGGAAC-3 and Mouse and Human QKI5 reverse primer 5-GATGGACACGCATATCGTG-3; Mouse and Human QKI6 forward primer 5-CTGTCATGCCAAACGGAAC-3 and Mouse and Human QKI6 reverse primer 5-CGTTGGGAAAGCCATAC-3; Mouse and Human QKI7 forward primer 5-CTGTCATGCCAAACGGAAC-3 and Mouse and Human QKI7 reverse primer 5-GACTGGCATTTCAATCCAC-3; Human GAPDH forward primer 5-TTCCAGGAGCGAGATCCCT-3 and Human GAPDH reverse primer 5-CACCCATGACGAACATGGG-3; Mouse Gapdh forward primer 5-GCACAGTCAAGGCCGAGAAT-3 and Mouse Gapdh reverse primer 5-GCCTTCTCCATGGTGGTGAA-3; Mouse L-Mag forward primer 5-TGCTCACCAGCATCCTCACG-3 and mouse L-Mag reverse primer 5-AGCAGCCTCCTCTCAGATCC-3.

### Electron microscopy

Mice were deeply anesthetized and then perfused with 4% glutaraldehyde, 0.1 M sodium cacodylate, and 3% sucrose, (pH 7.4). The tissues were then fixed in the same buffer. Samples were postfixed with osmium tetroxide, stained in 2% uranyl acetate, dehydrated, and embedded in Epon Beem Capsules. Coronal sections (80-nm thick) were prepared on a Leica UCT ultramicrotome and collected onto 20-mesh copper grids. Images were collected on JEOL L-1200EXII transmission electron microscope equipped with an 11-megapixel AMT digital camera. For the G-ratio, analyses were performed on three animals per genotype; a minimum of 50 myelinated axons was counted per animal. The G-ratio was obtained by determining the ratio of the circumference of the axon alone to the circumference of the fiber (axon and myelin) using GRatio for ImageJ^45^.

### Histopathology

Brain tissues were fixed in 10% formalin and embedded in paraffin per to standard procedures. Tissues were cut into 4-μm coronal sections, and slides were stained with anti-GFAP (Dako, Carpinteria, CA), anti-CD3 (Santa Cruz Biotechnology, Dallas, TX), anti-caspase 3 (cleaved caspase 3) (BioCare Medical, Concord, CA), or anti–Iba-1 (BioCare Medical). Slides labeled for GFAP and for IBA1 by IHC were imaged using an Aperio ScanScope (Leica Microsystems, Buffalo Grove, IL) with a 20x objective and analyzed using the corresponding ImageScope software. The area of the corpus callosum was traced in each slide to delineate the area of analysis. For GFAP, a positive pixel count algorithm was used to quantify the amount of positive labeling and results are expressed as the number of strongly positive pixels per square millimeter. For IBA1, a nuclear algorithm was used to count the number of positively labeled nuclei and results are expressed as the number of positive nuclei per square millimeter.

### Electrophysiological recordings

Procedures for estimating CAP amplitudes and conduction velocity were adopted from Crawford *et al*.^30^ with some modifications. Acute coronal mouse brain slices were prepared as follows. Mouse brains were isolated following decapitation and immediately placed in cold (4 °C) dissecting buffer containing 125 mM Choline-Cl, 2.5 mM KCl, 0.4 mM CaCl_2_, 6 mM MgCl_2_, 1.25 mM NaH2PO_4_, 26 mM NaHCO_3_, and glucose 20 (295–300 mOsm) under 95% O_2_/5% CO_2_. Coronal slices (400-μm thick) were cut in a Leica vibratome at 4 °C in the dissecting buffer. Slices were then transferred to oxygenated artificial cerebrospinal fluid (ACSF) containing 124 mM NaCl, 2.5 mM KCl, 1.25 mM NaH_2_PO_4_, 26 mM NaHCO_3_, 2 mM MgCl_2_, 2 mM CaCl_2_, 10 mM glucose at pH 7.4 and saturated with a 95% O_2_/5% CO_2_ gas mixture. Slices were allowed to equilibrate in this oxygenated ACSF for at least 1 h at room temperature prior to recording. Three to five slices per brain with midline-crossing segments of the corpus callosum were used for recording at room temperature.

### Compound action potentials

Corpus callosum fibers in coronal brain slices were stimulated using a concentric bipolar Pt/Ir electrode (diameters: outer pole, 200 μm; inner pole, 50 μm; FHC Inc., Bowdoin, ME). Recording electrodes were silver wires coated with chloride and positioned inside glass micropipettes filled with ACSF (resistance, ~3 mΩ). Stimulus pulses were constant current stimulus-isolated square waves delivered from an ISO-Flex^®^ stimulus isolator. For analyses of CAP amplitude, standardized input-output functions were generated for each slice by varying the intensity of stimulus pulses (100-μs duration, delivered at 0.2 Hz) in 250-μA steps from 0 to 4 mA for the short-latency negative CAP component. To enhance the signal-to-noise ratio, all quantitative electrophysiological analyses were conducted on waveforms that were the average of five successive sweeps. Evoked callosal CAPs were amplified, filtered (Bessel, 10 kHz), and digitized (200 KHz) using a Multiclamp 700B–1440A (Molecular Devices, Sunnyvale, CA) amplifier-digitizer combination, and stored on disk for offline analysis.

### Conduction velocity

The conduction velocity within the corpus callosum was estimated by changing the distance between the stimulating and recording electrodes from 1 to 2 mm while holding the stimulus intensity constant. Recordings were performed using the protocol described above for standard CAP measurements. The latencies to peak N1 and N2 components were plotted against the distance between the electrodes. The slope of the linear regression of the latency to peak represents velocity; thus, the latency to peak was used as an indirect measure of conduction velocity.

### *In vivo* Magnetic Resonance Imaging (MRI)

MRI was performed using a 7-Tesla Bruker Clinscan (Bruker BioSpin MRI GmbH, Germany) equipped with a Bruker 12S gradient (BGA12S) and a 2 channel phased-array surface coil. Animals were anesthetized using Isoflurane for the duration of the data acquisition. Turbo Spin Echo protocols (TR/TE = 1900–2500/39–42 ms) were used to acquire T2-weighted images (sagittal, transverse and coronal) using a matrix of 320 × 320 and field of view (FOV) of 25 × 25 mm. The volume of corpus callosum was obtained by manually segmenting the regions and computing volumes using Osirix (Pixmeo, Switzerland).

### Open-field test

The test arena (San Diego Instruments) consisted of a clear square arena with blue plastic floor measuring 16 inches ×16 inches with walls 16 inches tall. Testing was performed during the animal’s inactive phase under white light conditions. One hour before testing, mice were brought into the testing room and allowed to habituate. The activity of the mice was video recorded and scored using visual tracking software (CleverSys Inc. –Reston, VA). Locomotor activity was determined by allowing the mice to freely investigate the testing arena for 30 minutes. All surfaces were cleaned with 70% ethanol before and after each mouse.

### Hot Plate Test

Testing was performed during the animal’s inactive phase under white light conditions. One hour before testing, animals were brought to the testing rom and allowed to habituate. The hot plate test (Model 1440-D44 Columbus Instruments, Columbus, OH) was used to assess acute pain sensitivity to a thermal stimulus. The mice were submitted to a three testing trial at 50, 52.5 and 55 degrees Celsius. The latency of the first reaction of the hind and fore limbs (lick, shake, jump) is recorded. The maximum time that the mice were tested was 30 seconds to avoid possible tissue damage. Mice that did not react to heat after 30 seconds were removed. All surfaces were cleaned with 70% ethanol before and after each mouse. The hot plate data were from a single test on each mouse.

### Cell culture

The human T-lineage leukemia cell line CCRF-CEM was obtained from the American Type Culture Collection. Cells were cultured in RPMI-1640 medium containing 2 mM glutamine and 10% (vol/vol) FBS at 37 °C with 5% CO_2_.

### Stable short-hairpin RNA knock downs

CEM cells were infected with MISSION lentiviral transduction particles (Sigma-Aldrich, St. Louis, MO) produced from a library of sequence-verified shRNAs targeting human *MSH2* transcripts.

The following sequence of shRNA was used to knock down the *MSH2* gene:

5′-*CCGGATTCATGTTGCAGAGCTTGCTCTCGAGAGCAAGCTCTGCAACATGAATTTTTTG*-3′.

Nontarget shRNA control particles (SHC002V; Sigma-Aldrich) were also used. Individual cell clones were isolated in medium containing puromycin.

### Western blot analysis

Lysates from the CEM cell line or from mouse corpus callosum were separated by electrophoresis on a SDS-polyacrylamide gel. The proteins were then electroblotted onto a Hybond-P PVDF membrane. Protein expression was analyzed using the primary antibodies anti-GAPDH (sc-20357) and anti-MSH2 (NA27) anti MBP (AB980) purchased respectively from Santa Cruz biotechnology and from Calbiochem and Chemicon (Millipore). Horseradish peroxidase–conjugated secondary antibodies were purchased from Dako. The protein bands were quantified using ImageJ.

### Statistical analyses

Student’s t-test was used to compare the means in two different experimental conditions. Input-output electrophysiological function data were analyzed using two-way analysis of variance (ANOVA) (genotype and stimulus strength), and latencies to peak were compared using two-way (genotype and distance between the two electrodes), repeated-measures ANOVA.

Effect sizes were assessed by estimating the mean differences and the accuracy of the estimate is assessed by 95% confidence interval.

Analyses were done using SAS (Cary, NC), R (The R Project http://www.r-project.org/) and SigmaPlot 11.0.

## Additional Information

**How to cite this article**: Diouf, B. *et al. Msh2* deficiency leads to dysmyelination of the corpus callosum, impaired locomotion, and altered sensory function in mice. *Sci. Rep.* **6**, 30757; doi: 10.1038/srep30757 (2016).

## Supplementary Material

Supplementary Information
