# Supplementary material for: Msh2 deficiency leads to dysmyelination of the corpus callosum, impaired locomotion, and altered sensory function in mice
Source: Sci Rep. 2016 Aug 1;6:30757. doi: 10.1038/srep30757 (PMC4967871; doi:10.1038/srep30757)

***Msh2* deficiency leads to dysmyelination of the corpus callosum, impaired locomotion, and altered sensory function in mice.**

Barthelemy Diouf[1], Prakash Devaraju[2], Laura J. Janke[3], Yiping Fan[4], Sharon Frase[5], Donnie Eddins[2], Jennifer L. Peters[5], Jieun Kim[6], Deqing Pei[7], Cheng Cheng[7], Stanislav S. Zakharenko[2], and William E. Evans[1*]

[1]Hematological Malignancies Program and Department of Pharmaceutical Sciences, St. Jude Children's Research Hospital, Memphis, 38105, USA; [2]Departmentof Developmental Neurobiology, St. Jude Children's Research Hospital, Memphis, 38105, USA; [3]Department of Pathology, St. Jude Children's Research Hospital, Memphis, 38105, USA; [4]Department of Computational Biology, St. Jude Children's Research Hospital, Memphis, 38105, USA; [5]Cell and Tissue Imaging Resource, St. Jude Children's Research Hospital, Memphis, 38105, USA; [6]Small Animal Imaging Center, St. Jude Children's Research Hospital, Memphis 38105, USA ; [7]Department of Biostatistics, St. Jude Children's Research Hospital, Memphis 38105, USA.

*Corresponding author : Dr. William E. Evans
Administration, MS 272, St. Jude Children's Research Hospital
262 Danny Thomas Place, Memphis, TN 38105, USA
Phone: (901) 495-3301
Fax: (901) 525-6869
Email: william.evans@stjude.org

Supplementary Information.

Supplementary Figure S1. Electron microscopy of axons in the corpus callosum.
Histograms showed no difference in axon diameter in the *Msh2*$^{-/-}$ compared to wild-type Mice in the corpus callosum. Quantification was performed from 50 axons in each mouse. Error bars represent SD. N=3 mice for each genotype.

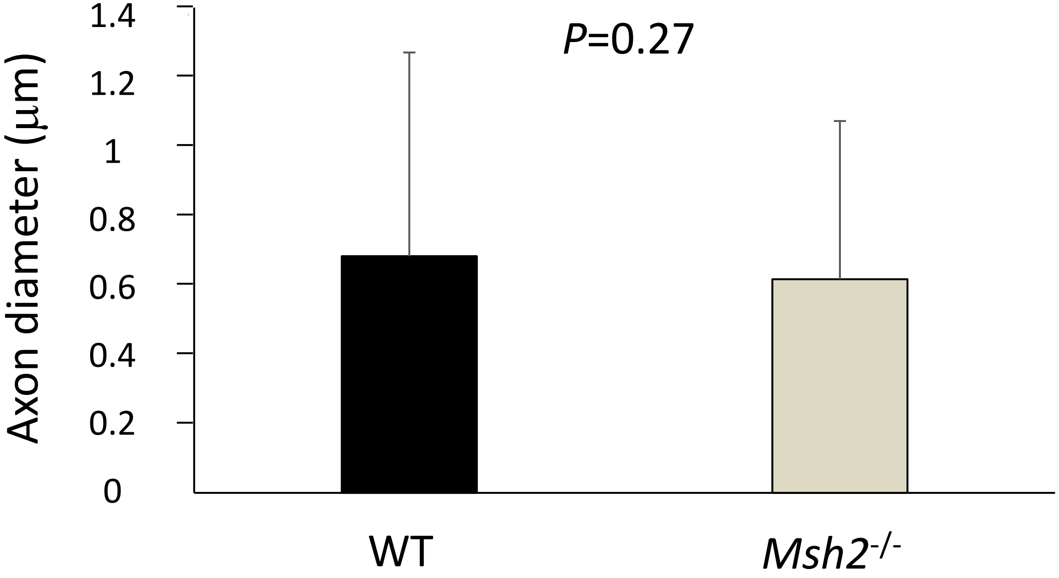

Supplementary Figure S2. Immunohistochemical labeling using anti CD3 antibody.
Paraffin sections show no infiltration of CD3+ T cells in the corpus callosum of the wild-type (a) and the *Msh2*$^{-/-}$(b) mice. Representative images of n=4 mice for each genotype are shown.

a b

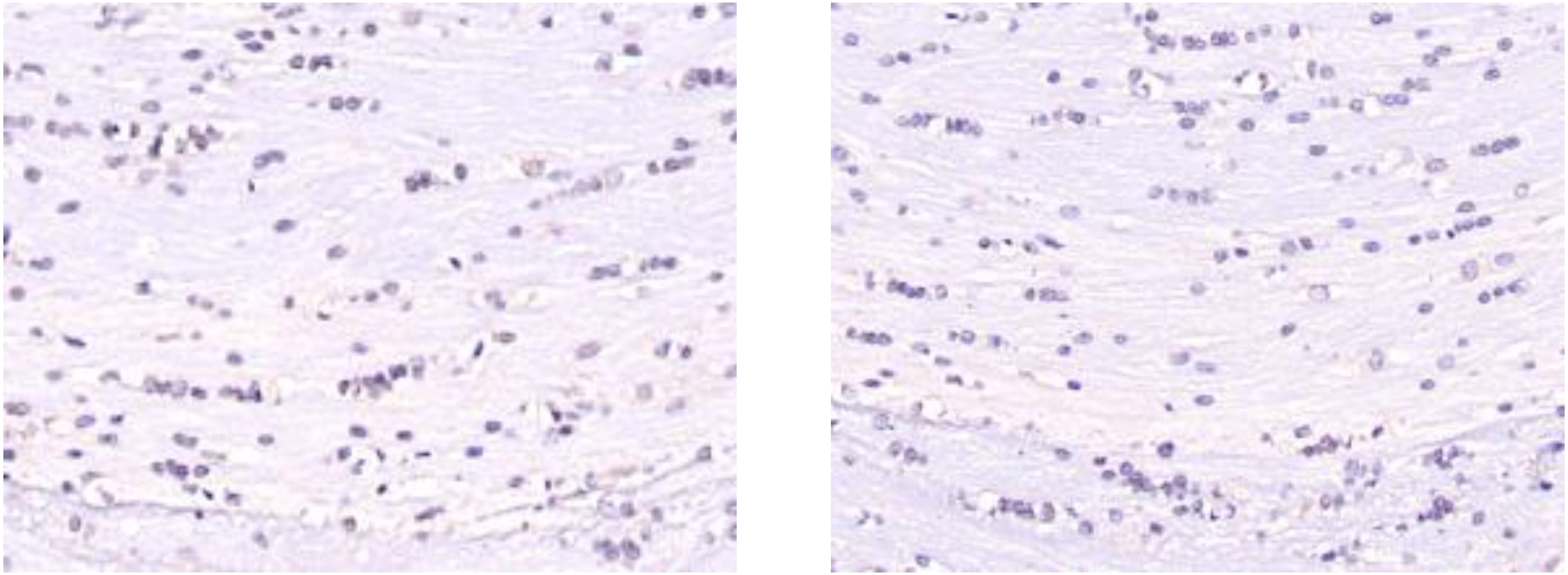

Supplementary Figure S3. Immunohistochemical labeling using anti GFAP antibody.
Paraffin sections show no difference in GFAP staining in the corpus callosum of the wild-type (a) and the *Msh2*$^{-/-}$(b) mice. Representative images of n=4 mice for each genotype are shown.

a b

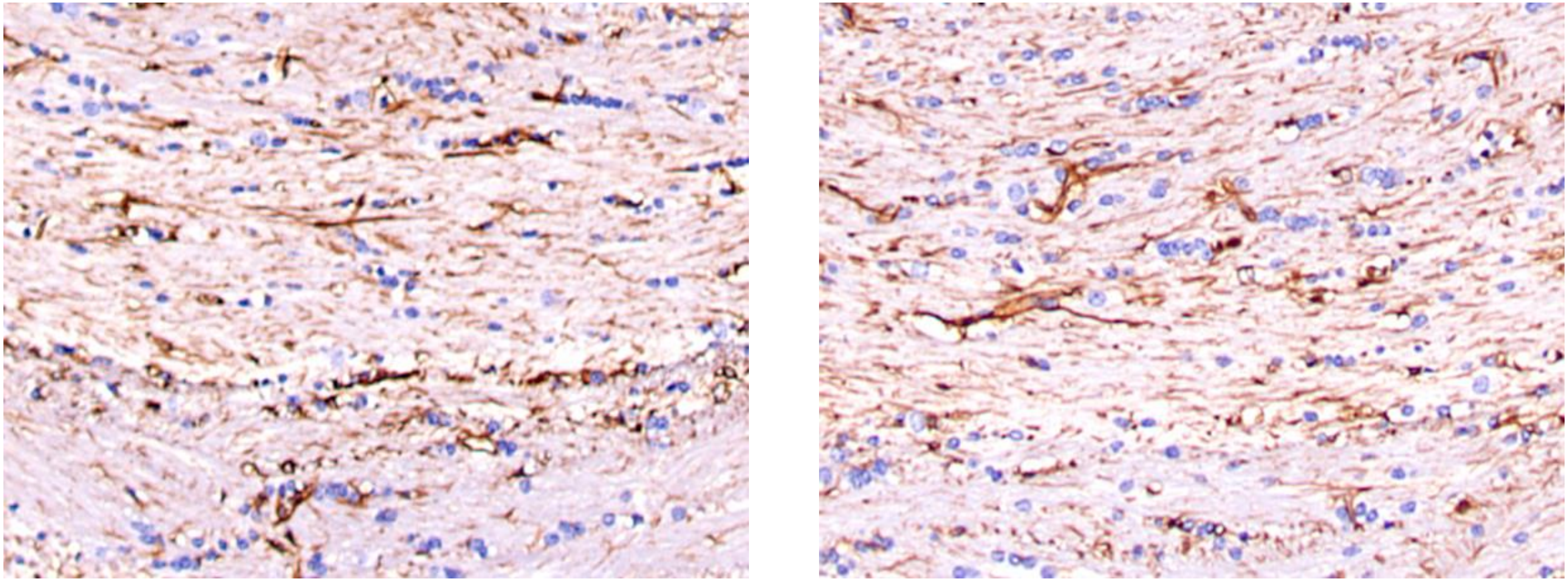

Supplementary Figure S4. Immunohistochemical labeling using anti Iba-1 antibody.
Immunohistological labeling of Iba-1 reveals no accumulation of activated microglia cells in the callosum of wild-type (a) and *Msh2*$^{-/-}$(b) mice. Representative images of n=4 mice for each genotype are shown.

a b

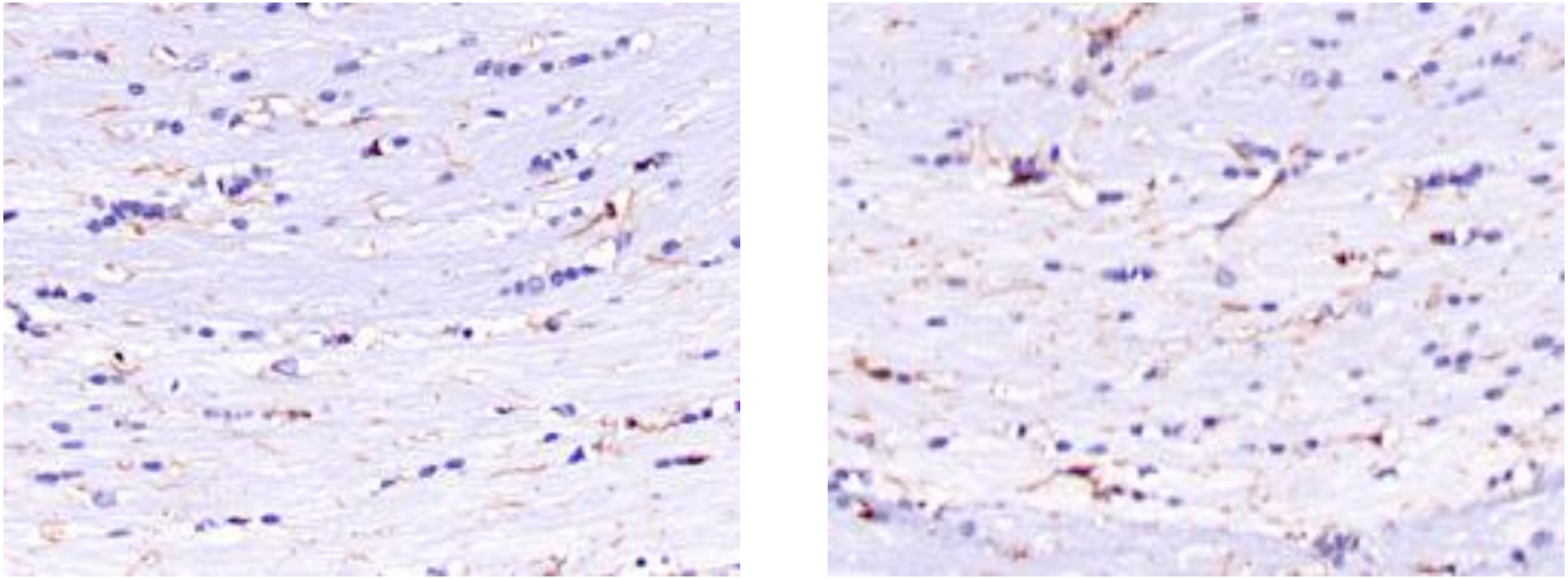

Supplementary Figure S5. Immunohistochemical labeling using anti anti-cleaved caspase 3 antibody.

Coronary sections of the mice brain paraffin embedded and labeled with anti-cleaved Caspase 3 antibody show not increased apoptosis in the Corpus callosum of the wild-type (a) *Msh2*$^{-/-}$(b) mice. Representative images of n= 4 mice for each genotype are shown.

a

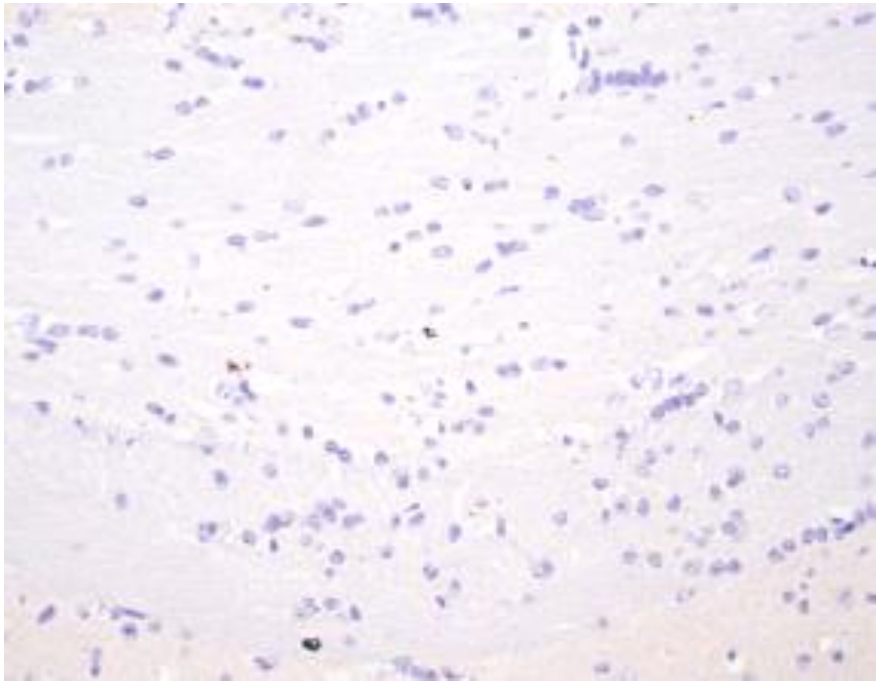

b

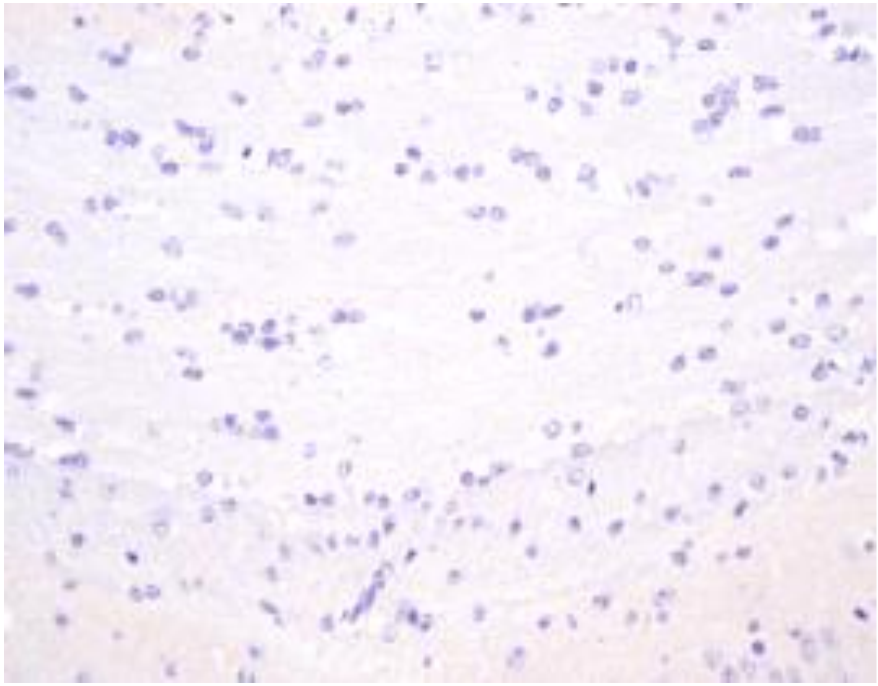

Supplementary Figure S6. MBP expression in the corpus callosum.

Western blot analyses of MBP expression show lower expression of the different isoforms of MBP in the corpus callosum of *Msh2*$^{-/-}$ mice compared to wild-type mice (n=3 for each genotype). Representative images of each genotype are shown. GAPDH was used as loading control.

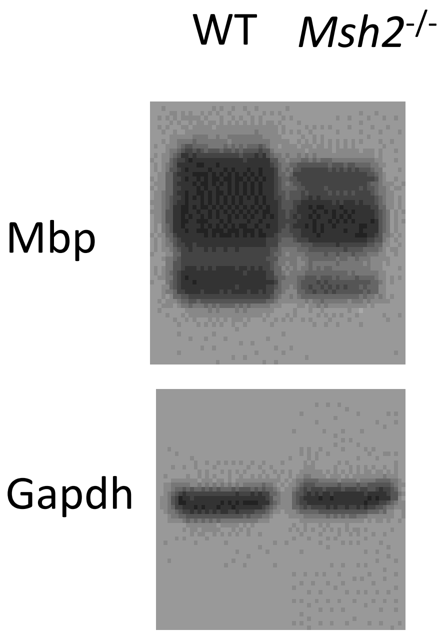

Supplementary Figure S7. *L-Mag* expression in the corpus callosum.

mRNA expression of *L-Mag* in the corpus callosum was determined. Quantification of *L-Mag* mRNA levels in the corpus callosum normalized to Gapdh signal shows low expression of *L-Mag* in the *Msh2*$^{-/-}$ mice compared to WT mice. Error bars represent SD (n=3 for each genotype). (p<0.001, 95% confidence interval of difference in mean [- 0.52 to -0.44])

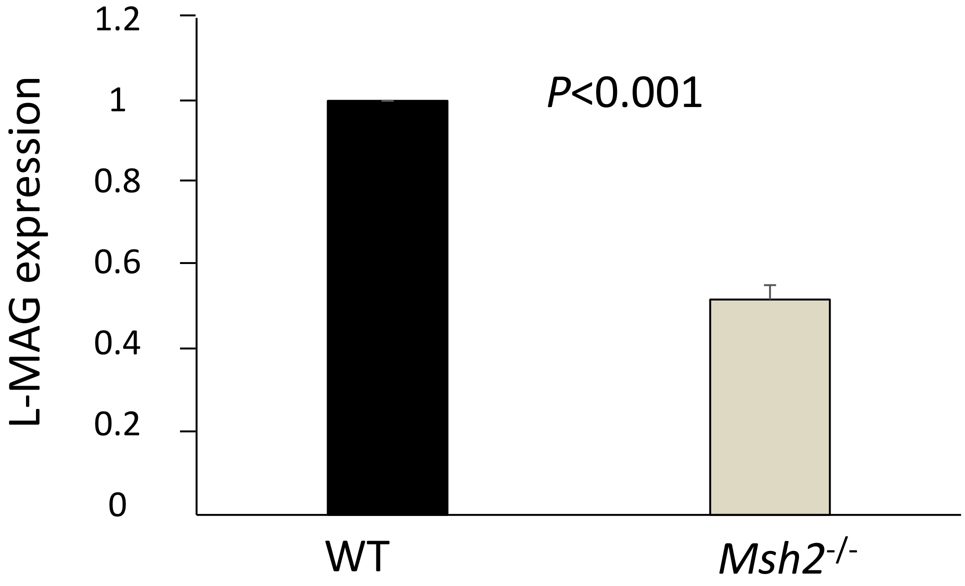

Supplementary Figure S8. ***In vivo*** **MRI.**

Corpus callosum volumes (normalized to Brain volume) determined by MRI were smaller in the *Msh2*$^{-/-}$ mice (b) compared to the wild-type mice (a). Representative images of n=4 mice for each genotype are shown. The corpus callosum is indicated in green.

a b

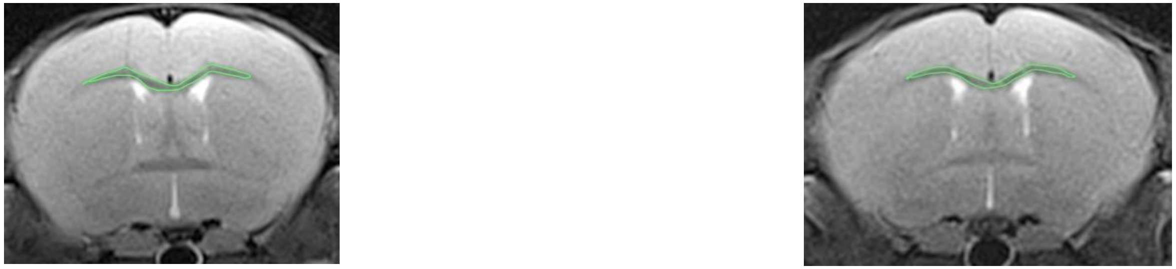

Supplementary Figure S9. *Full-length blots*.
(a) and (b) represent Full-length blots of figure 1a and figure 1e respectively.

a

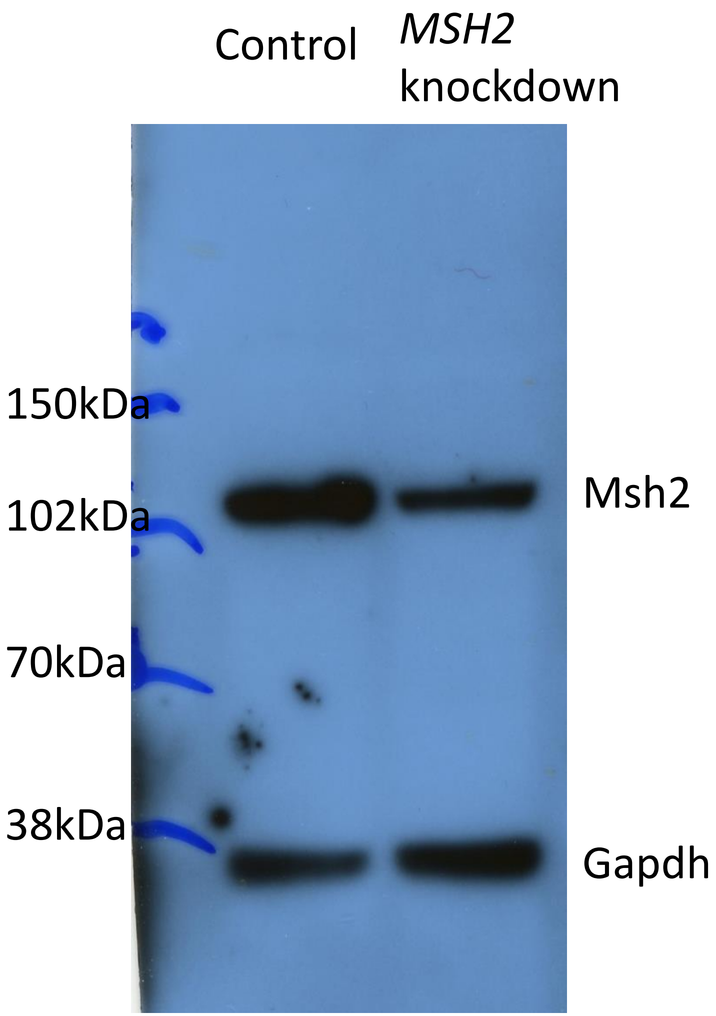

b

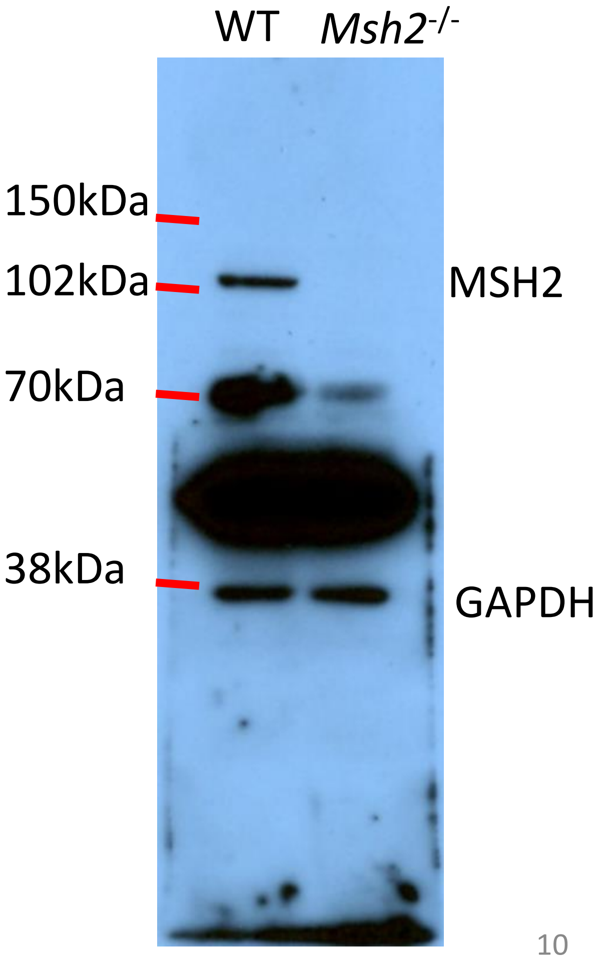

Supplement: Supplementary Information [file srep30757-s1.pdf]
